# High-Resolution Imaging of Dendrimers Used in Drug Delivery via Scanning Probe Microscopy

**DOI:** 10.1155/2011/254095

**Published:** 2011-06-13

**Authors:** Lifang Shi, Christopher J. Fleming, Shawn L. Riechers, Nai-Ning Yin, Juntao Luo, Kit S. Lam, Gang-yu Liu

**Affiliations:** ^1^Department of Chemistry, University of California, Davis, CA 95616, USA; ^2^Department of Biochemistry and Molecular Medicine, UCD Cancer Center, University of California, Davis, Sacramento, CA 95817, USA; ^3^Division of Hematology and Oncology, Internal Medicine, UCD Cancer Center, University of California, Davis, Sacramento, CA 95817, USA

## Abstract

Dendrimers and telodendrimer micelles represent two new classes of vehicles for drug delivery that have attracted much attention recently. Their structural characterization at the molecular and submolecular level remains a challenge due to the difficulties in reaching high resolution when imaging small particles in their native media. This investigation offers a new approach towards this challenge, using scanning tunneling microscopy (STM) and atomic force microscopy (AFM). By using new sample preparation protocols, this work demonstrates that (a) intramolecular features such as drug molecules and dendrimer termini can be resolved; and (b) telodendrimer micelles can be immobilized on the surface without compromising structural integrity, and as such, high resolution AFM imaging may be performed to attain 3D information. This high-resolution structural information should enhance our knowledge of the nanocarrier structure and nanocarrier-drug interaction and, therefore, facilitate design and optimization of the efficiency in drug delivery.

## 1. Introduction

Using nanoparticles composed of polymers or assemblies of amphiphilic molecules as drug delivery vehicles have attracted much attention in the recent quest for drug delivery [1–3]. Among them, many dendrimers have been synthesized with a great degree of control in the synthesis of the designed structure [4, 5]. Dendrimers exhibit great promise as nanocarriers for efficient drug delivery due to researchers' ability to control their size (e.g., 1 nm to 100 nm) through the variation of iteration cycles and to implement surface and intramolecular functionalities designed to carry or trap desired drug molecules through covalent, hydrophobic, ionic, or hydrogen-bonding interactions [1, 6–9]. Successful examples have been reported, such as the increase in solubility and *in vivo* compatibility of non-steroidal anti-inflammatory drugs (NSAIDs) using functionalized dendrimer carriers [10–14]. Specifically, the combination of indomethacin with poly(amidoamine) (PAMAM) dendrimers resulted in enhanced *in vivo* pharmacokinetic performance over indomethacin alone [15]. 

Complimentary to the “hard” dendrimers discussed above, “soft” nanostructures, such as nanomicelles, made by assembly of biocompatible telodendrimers (e.g., a linear poly(ethylene glycol)- (PEG-) block-dendritic oligomers of cholic acid (CA)) in aqueous conditions were also developed recently [16–21]. These nanomicelles are highly flexible, and as such, they exhibit the advantage for *in vivo *movement. Since polymer molecules are the basic unit within micelles, multifunctionalities may be implemented for individual molecules, and size may be tuned (e.g., *d* = 15–300 nm) by varying the conditions of assembly. The amphiphilicity enables the incorporation of hydrophobic drugs such paclitaxel (PTX) enclosed inside the micelles, with a load capacity as high as 7.3 mg/mL [16]. The *in vitro* anticancer activity of PTX loaded PEG^5k^-CA_8_ micelles have been performed on human ovarian clear cell carcinoma cells (ES-2) and firefly luciferase-expressing ovarian adenocarcinoma cell lines (SKOV3-luc-D3). PTX-loaded PEG^5k^-CA_8_ micelles exhibited equivalent cytotoxic activity *in vitro* compared with the clinical formulations of PTX, such as Taxol and Abraxane [17]. *In vivo *antitumor efficacy of PTX loaded PEG^5k^-CA_8_ micelles have been tested in nude mice bearing human SKOV3-lue ovarian cancer xenograft, where the results indicated that this vehicle could deliver PTX preferentially to tumor sites via enhanced permeation and retention (EPR) effect, and thus exhibits superior *in vivo* anticancer effect overall in animal models, compared to Taxol and Abraxane [16, 17]. 

 To enhance the efficiency of drug delivery, knowledge of the nanocarrier structure and nanocarrier-drug interaction is critical for their design and optimization. In the case of dendrimer systems, the location and the binding of drug molecules to dendrimers are particularly important, as the outcome is directly related to loading capacity and release behavior. While macroscopic information such as solubility [12–14] and spectroscopy [22] are available, little is known at the molecular level. Despite the prediction by molecular dynamics simulations [23–25] that drug molecules may attach to both the exterior and interior of dendrimers, the direct evidence from experiments is still lacking due to difficulties in visualizing intramolecular structures of dendrimers. Scanning tunneling microscopy (STM), due to its high spatial resolution, offers a promising solution to this challenge [26]. The highest spatial resolution is typically reported for conductive and semiconductive systems, reaching the submolecular level [27]. Using metal ion coordination [28, 29], we extended the high-resolution capability of STM to dendrimers in this investigation, resolving individual indomethacin molecules at the dendrimer exterior. 

 In the case of telodendrimer micelles, dynamic light scattering (DLS) allows the average diameter and distribution to be determined in the solution phase [16, 17]. Individual micelles may be visualized using cryotransmission electron microscopy (cryo-TEM) upon freezing of the samples. The use of cryo-TEM is complicated, as the micelles are no longer in their natural environment [30]. A much simpler technique, atomic force microscopy (AFM), could offer some remedy to this pursuit. AFM offers high spatial resolution and versatility of imaging in various media, including micelle formation media and physiological buffers [31–33].

In this study, we have tested the feasibility and demonstrated the proof-of-concept of using scanning probe microscopy to image PTX-loaded thiol modified telodendrimer micelles, HS-PEG^5k^-CA_8_ (“5k”, represents the molecular weight of PEG, and “8” indicates the number of CA subunits in the telodendrimer), in aqueous media where micelles form. The results are very encouraging: individual micelles are clearly visualized, from which we can extract the size and geometry of micelles in correlation with the conditions of assembly. The difference between native and drug carrying micelles is clearly visible under AFM, from which the drug carrying capacity can be estimated. In addition, the knowledge of the geometry and size of individual micelles facilitates our understanding of their efficacy and further optimization.

## 2. Materials and Methods

### 2.1. Materials

Paclitaxel was purchased from AK scientific Inc. 4th generation hydroxyl-terminated poly(amidoamine) dendrimers, G4 PAMAM-OH (98% purity, 10% by weight in methanol), and 1-(4-Chlorobenzoyl)-5-methoxy-2-methyl-3-indoleacetic acid, commonly known as indomethacin (≥99.0%), were purchased from Sigma-Aldrich and used without further purification. 1-adamantanethiol (AD, 95% purity) and *n*-octanethiol (C_8_, 98% purity) were purchased from Sigma-Aldrich and used as received. 200 proof ethanol (99.99% purity) was purchased from Gold Shield Chemical Co. K_2_PtCl_4_ (min. 42.4% Pt, Alfa Aesar) was used as received. Ultrapure water (≥8 MΩ·cm) was obtained using a Millipore Milli-Q filtration system. Ultrapure N_2_ (≥98%, Air Gas Co.) and H_2_ (99.99%, Praxair, Inc.) were used for drying and flaming, respectively. STM tips were made from W wire (*d* = 0.010 in, 99.95%, California Fine Wire Co.). Epoxy glue (Epo-tek 377) was purchased from Epoxy Technology.

### 2.2. Synthesis of Thiol Functionalized Telodendrimer HS-PEG^5k^-CA_8_

BocNH-PEG^5k^-CA_8_ was synthesized following the established procedure [16]. The Boc protecting group was removed via the treatment with 50% of trifluoroacetic acid (TFA) in dichloromethane (DCM) for 30 min, and then, the majority of solvent was removed by blowing nitrogen. The polymer was precipitated by washing three times with cold ether. S-Trityl-beta-mercaptopropionic acid (2 equ.) was coupled on the amino groups on the terminal end of telodendrimer using hydroxybenzotriazole (HOBt, 2 equ.) and diisopropylcarbodiimide (DIC, equ.) as coupling reagents overnight. The telodendrimer was precipitated and washed by cold ether and was treated with 50% TFA in DCM for 30 min, then the majority of solvent was removed by blowing nitrogen. The telodendrimer was precipitated and washed by cold ether and dissolved in water. The telodendrimer solution was filtered and then dialyzed against 4 L water in a dialysis tube with molecular weight cut-off (MWCO) of 3.5 KDa; reservoir water was refreshed completely four times in 24 h. Finally, the telodendrimer was lyophilized. The molecular weight of the telodendrimer was detected by matrix-assisted laser desorption ionization—time of flight mass spectrometry (MALDI-TOF MS) and nuclear magnetic resonance (NMR) spectrometry. The thiol group was detected by Ellman's assay. The synthesized HS-PEG^5k^-CA_8_ telodendrimer was kept in desiccators before use.

### 2.3. Preparation of Gold Thin Films

Au(111) thin films were prepared via thermal evaporation of Au onto freshly cleaved mica (0001) in a high-vacuum evaporator (Denton Vacuum, Model 502-A) [34]. The substrate mica was heated via two quartz lamps to 350°C under a base pressure of 2 × 10^−7^ torr. The evaporation rate was 0.3 nm/sec and the final thickness of Au films was 150 nm. After evaporation, the Au was thermally annealed in situ at 375°C for 30–60 min to increase the size of the Au(111) terraces. After annealing, the Au film was allowed to cool for ≥5 hr. under vacuum. Upon removal, the Au films were stored in a sealed glass container.

### 2.4. Preparation of 1-Adamantanethiol Self-Assembled Monolayers (SAMs)

The gold films described above were used to prepare ultraflat gold films on glass substrates following a method reported previously [35]. Briefly, the gold films were annealed in an H_2_ flame in order to coalesce the gold grains on the mica. Then, the gold thin film was cooled in air to room temperature. A small droplet of epoxy glue was applied to each of the dry glass substrates (coverslips). The coverslips were then placed on the gold substrate with the glue attached side facing down. The glue was then cured at 150°C overnight. After removal from oven, the glass coverslip with gold thin film were peeled off from the mica substrate. The 1-adamantanethiol (AD) SAMs were prepared by immersing the gold films in a 10 mM ethanolic AD solution at room temperature for 24 hours [36]. The AD SAM on gold was rinsed first with ethanol, and then with Milli Q water, before the deposition of the loaded or unloaded micelles.

### 2.5. Loading of PTX into HS-PEG^5k^-CA_8_ Micelles and Characterization

6 mg of PTX and 20 mg of HS-PEG^5k^-CA_8_ were dissolved in 3 mL of chloroform in a 10 mL single neck flask to form a homogeneous solution. The solvent was removed by rotavaporation, and the sample was further dried on high vacuum pump for 30 min. Then, 1 mL of phosphate buffered saline (PBS) was added into the flask to disperse the solid film via vortex and further sonication for 30 min to yield a homogenous micelle solution. The particle sizes of the micelles before and after PTX loading were measured with DLS Zetatrac (Microtrac) to be 16 nm and 23 nm, respectively. The drug loading capacity was measured using high-performance liquid chromatography (HPLC) calibrated with PTX solutions in dimethyl sulfoxide (DMSO) with known concentrations.

### 2.6. AFM Imaging

AFM measurements of micelles and dendrimers were performed on a MFP3D AFM (Asylum Research Inc., Santa Barbara, Calif, USA). When imaging HS-PEG^5k^-CA_8_ and PTX-loaded HS-PEG^5k^-CA_8_ micelles in SAMs, tapping mode was utilized in aqueous solution. The probe is a MSNL-10 silicon cantilever (Veeco, Camarillo, Calif, USA) with a force constant of *k *= 0.1 N/m. During AFM tapping, the cantilever was modulated by a driving frequency of 68 kHz and an amplitude range from 0.30 to 0.71 V, with damping from 30 to 70%. When imaging PAMAM dendrimers, a silicon cantilever (AC-240, Olympus) was used. The probe has a force constant of *k* = 1.0 N/m as measured by thermal noise method. During tapping mode imaging, the cantilever was modulated by a driving frequency of 74 kHz and amplitude of 67.0 nm (0.63 V), with the damping set to 85%. For displacing adsorbates such as dendrimers or alkanethiolates, tips were placed in contact with the surface with increasing load beyond threshold [28, 29]. Data display and analysis were conducted using MFP-3D's software package written on Igor Pro platform (Wavemetrics). The surface coverage of micelle was calculated based on AFM topography images.

### 2.7. STM Imaging

The STM has a walker-type scanner (UHV 300, RHK Technologies, Inc.) and was used under ambient conditions for this investigation. The STM tips were cut W wires which were electrochemically etched at 2.0 V in 3.0 M NaOH solutions. A homemade potentiostat monitored the etching process [34, 37]. All STM images were acquired in constant current mode with typical bias voltages ranging from 0.3 to 0.7 V and tunneling currents from 5 to 25 pA. The piezoelectric scanners were calibrated laterally using a decanethiol SAM (lattice constant = 0.50 nm) and vertically using a Au(111) single atomic step (0.235 nm).

## 3. Results and Discussion

### 3.1. Immobilization of Telodendrimer Micelles into SAMs for AFM Imaging

**Scheme 1:**
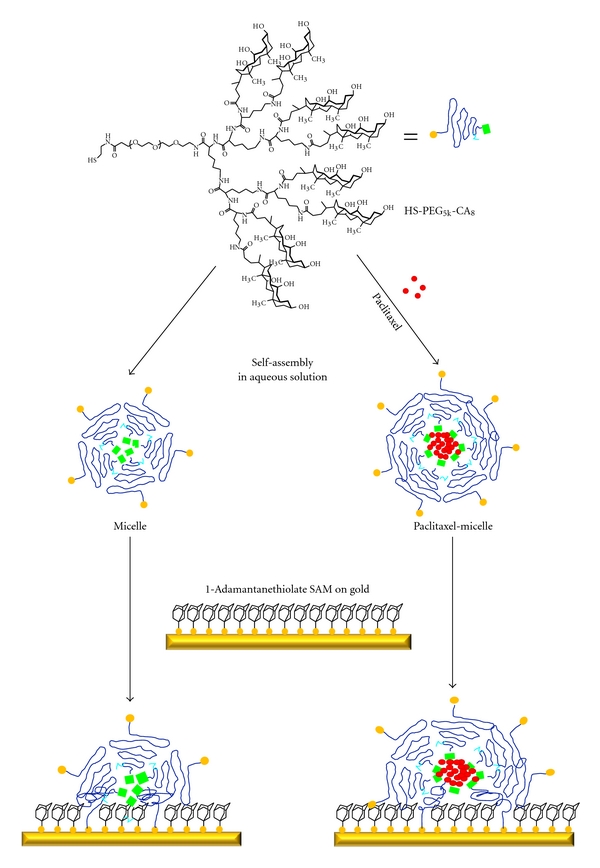
Schematic of surface immobilization of unloaded and paclitaxel loaded HS-PEG^5k^-CA_8_ micelles on Au surfaces.

 For structural characterization via AFM, micelles must be immobilized on surface supports. Immobilized drug delivery vehicles are the key component in therapies using patches [38]. A potential application of immobilized PTX-loaded micelles on surfaces is the development of a new type of PTX eluting stent [39]. The procedure of immobilization of micelles onto gold surfaces is shown in Scheme 1. HS-PEG^5k^-CA_8_ telodendrimer is soluble in water and self-assembles into micelles. PTX is loaded into the micelle via a procedure of solvent evaporation followed by the aqueous dispersion of micelles [40]. 

 In order to maintain the integrity of micelles on solid surface, gold surfaces were covered by SAMs of AD. The use of AD is based mainly on two considerations: (a) SAMs provide a buffer to dampen collisions and allow full contact between micelles and gold surfaces and (b) AD can be exchanged by alkane thiol functionalities to enable micelles to anchor onto gold surfaces. As illustrated in Scheme 1, micelles are formed instantly via the self-assembly of telodendrimers dissolved in aqueous solution. The critical micelle concentration of micelles was 5.3 *μ*M. The micelles have noncharged surfaces, the Zeta-potential was measured close to zero [21]. AD SAMs on gold were soaked in micelle solutions, 0.5 mg/mL, for 20 min. This short exposure resulted in 15.3% surface coverage of micelles on the gold surface. In the case of PTX-loaded micelles, a concentration of 26.4 mg/mL (weight ratio as 6.4 mg PTX: 20 mg HS-PEG^5k^-CA_8_) was used and the exposure time was typically 1 hour. This led to 29.0% surface coverage of PTX loaded micelle on the gold surface. After deposition, the samples were rinsed with Milli Q water and kept in the water solution before AFM measurement.

### 3.2. AFM Enables Visualization Telodendrimer Micelles in their Native Media and Detection of Changes upon Uptake of PTX

**Figure 1:**
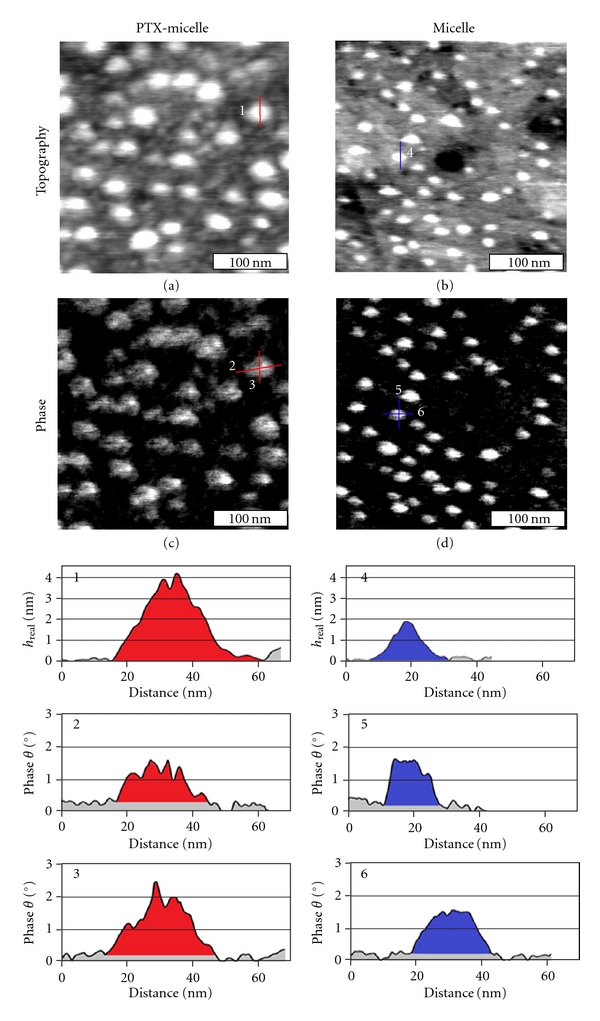
AFM characterization of paclitaxel- (PTX-) loaded HS-PEG^5k^-CA_8_ micelles and unloaded HS-PEG^5k^-CA_8_ micelle on gold substrate in aqueous solution. The first column is AFM topography (a), phase image (b) and corresponding cursors of PTX-loaded micelle. AFM topography (c), phase image (d), and corresponding cursors of unloaded micelle are shown in second column. In cursor profiles, areas indicated with red, blue, and grey are relative to PTX-loaded micelle, unloaded micelle and AD SAM, respectively. Height, short axis, and long axis of PTX-loaded micelle extracted from cursors profiles 1, 2, and 3. Height, short axis, and long axis of unloaded micelle extracted from cursors profiles 4, 5, and 6.

 Upon immobilization, AFM imaging is carried out in water media. To attain accurate measurements in 3D without significant deformation, tapping mode is utilized, from which height is extracted from topographic images, and lateral boundaries are well defined from phase images. The AFM images in Figure 1 indicate that all micelles, PTX-loaded or unloaded, maintain the geometry of elliptical cap geometry. Figure  1(a) is a 300 × 300 nm^2^ AFM topography image of PTX-loaded micelles on ultraflat Au. Each bright protrusion is a single PTX-loaded micelle. The height of a typical PTX-loaded micelle, as shown in cursor 1, is 4.0 nm, measured from the lowest point in the local surroundings to the apex of the micelle. Its lateral boundaries are clearly visible from the AFM phase image shown in Figure  1(c). The lateral dimensions are 28.1 nm and 33.0 nm for short axis and long axis, respectively, as shown in cursors 2 and 3. Among the 49 PTX-loaded micelles measured, the average 3D dimensions (long axis “a”, short axis “b”, and height “h”) are 31.8 ± 4.3 nm, 25.6 ± 3.2 nm, and 4.6 ± 0.7 nm, respectively. As a comparison, Figure  1(b) is a 300 × 300 nm^2^ AFM topography image of original micelles on ultraflat Au. The measured height of the unloaded micelle is 1.9 nm, as shown in cursor 4. Figure 1(d) is the phase image of unloaded micelle, from which the lateral boundaries are clearly visible. The short and long axis of the unloaded micelle is 17.3 nm and 25.2 nm, respectively, as shown in cursors 5 and 6. Among the 50 unloaded micelles measured, the *a*, *b*, and *h* measure 23.7 ± 2.4 nm, 17.2 ± 2.3 nm, and 1.8 ± 0.2 nm, respectively. 

 The volume, *V*, of each micelle can be calculated using the simple geometric formula: *V* = (1/6*πh*)(3/4*ab* + *h* ^2^)  [28]. From Figures  1(a) and 1(c), the height of PTX-loaded micelle is 4.0 nm, the lateral dimensions are *a* = 33.0 nm, *b* = 28.1 nm, thus *V* = 1490.1 nm^3^. From Figures  1(b) and 1(d), the unloaded micelle, measures *a* = 25.2 nm, *b* = 17.3 nm, and *h* = 1.9 nm, which corresponds to *V* = 328.9 nm^3^. The average volume of PTX-loaded micelle and unloaded micelle is 1475.8 ± 396.2 nm^3^ and 295.1 ± 62.6 nm^3^, respectively. PTX-loaded micelle exhibits a larger volume than unloaded micelle. Our Investigations also reveal that the amounts of PTX uptake affect the volume of micelles. 

 By assuming that the micelle has a spherical shape in water solution, we can estimate the diameter of micelles in solution based on *V* = 4/3*π*(*D*/2)^3^. Here, *V* is volume and *D* is the diameter. The volume of a typical PTX-loaded micelle in Figure  1(a) is 1490.1 nm^3^, the corresponding diameter is 14.2 nm. Among the 49 PTX-loaded micelles measured, the average diameter is 14.2 ± 1.2 nm. The volume of a typical unloaded micelle in Figure  1(b) is 328.9 nm^3^, the corresponding diameter is 8.6 nm. Among the 50 unloaded micelles measured, the average diameter is 8.2 ± 0.6 nm. The diameter of PTX-loaded and unloaded micelle obtained from the dynamic light scatting (DLS) measurement is 23 ± 8 and 16 ± 4 nm, respectively [16]. One notes that the size of adsorbed micelles as determined from AFM experiments is smaller than the corresponding diameter measured from the DLS in solution. While DLS gives the averaged hydrodynamic radius of the scattering particles, AFM provides true 3D measurements of individual micelle. The dimensions extracted from AFM measurements more truly reflect the true geometry of the micelles, In addition, it is difficult to reach high accuracy if the particle is very small and nonspherical, for example, <10 nm [41], while AFM does not have such a limitation. 

 As a bonus, we can estimate the number of PTX molecules based on the volume measurements from AFM. The numbers of PTX (*N*_ptx_) and telodendrimers (*N*_telo_) are estimated from two equations below, (a) assuming that the volume of individual components are conserved, based on Connolly solvent-excluded volume [42] using Chem3D Software, using telodendrimer volume of 13.13 nm^3^, and PTX being 0.754 nm^3^; (b) the mixing follows 7.5: 2.1 = PTX: telodendrimers. Therefore, for a typical PTX loaded micelle in Figure  1(a), the volume is 1490.1 nm^3^

(1) $$\frac{N_{\text{ptx}}}{N_{\text{telo}}}=\frac{7.5}{2.1},$$

(2) $$N_{\text{ptx}}\times 0.754+N_{\text{telo}}\times 13.13=1490.1.$$

Solving (2) with (1), *N*_ptx_ = 336, while *N*_telo_ = 94. For a typical micelle indicated in Figure  1(b), there are 25 telodendrimer units. Within a typical PTX-loaded micelle as shown in Figure  1(a), there are 336 PTX molecules and 94 telodendrimers. The increase in overall size upon PTX loading is likely due to the increase in the number of the telodendrimer molecules within individual micelles. The hydrophobicity of PTX may require larger number of amphiphilic telodendrimers to enclose them inside micelles for overall reduced enthalpy [43]. 

Taken collectively, AFM provides an alternative and more accurate approach to measure the geometry and size of individual drug delivery vehicles. Even for soft systems such as HS-PEG^5k^-CA_8_ micelles, AFM images may be attained in their native media. This versatility of imaging in water media at a designed temperature allows direct comparison before and after loading or uptake of drugs. In addition, the accuracy enables quantification, such as the determination of height, lateral dimension, volume, and number of drugs enclosed. Therefore, we encourage researchers to consider the application of AFM in determination of the size and geometry of drug-carrying vehicles in the various synthetic and drug-loading steps.

### 3.3. Preparation and Immobilization of PAMAM Dendrimers for High-Resolution Imaging

**Scheme 2:**
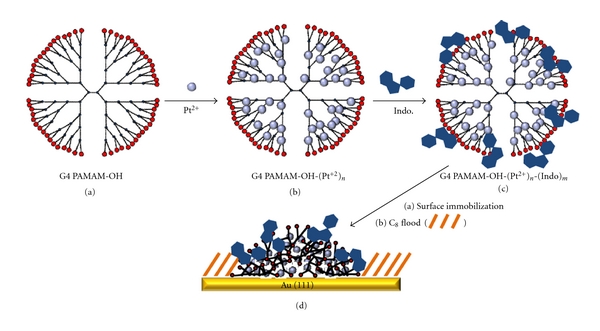
Methodology for the metal ion coordination, drug loading, surface immobilization, and passivation of G4 PAMAM-OH dendrimers. Dendrimers in solution (a) are doped with Pt^2+^ ions (b). Indomethacin is then added to the solution (c). The conductive, drug-loaded dendrimers are then exposed to Au(111) (a) followed by C_8_ flooding, (b), to obtain the surface-immobilized G4 PAMAM-OH-(Pt^2+^)_*n*_-(Indo)_*m*_.

To visualize intramolecular structure of PAMAM dendrimers using STM, two key steps are involved, surface immobilization and introduction of metal ions to enable the transport of STM current [28]. Detailed protocols for dendrimers have been discussed previously [28, 29]. For indomethacin carrying dendrimers, first, G4 PAMAM-OH dendrimer solutions were made by diluting aliquots of the methanol-based stock solutions to 12.5 *μ*M aqueous solutions. Second, as illustrated in Scheme 2, K_2_PtCl_4_ was then added to achieve a molar ratio of 1 : 120, dendrimer: Pt^2+^. The ratios were guided by the number of tertiary amines (dendrimer branch points) within individual dendrimers, for example; G4 has 62 tertiary amines. Once mixed, the solution was kept at room temperature for 3–5 days, allowing sufficient time for Pt(II)-amine coordination within dendrimers [44]. Third, indomethacin was weighed and then directly added to reach a final stoichiometry of 1 : 120 dendrimer:indomethacin molar ratio to maximize the potential for interaction between the drug and the dendrimer −OH termini and available tertiary amines. Final dendrimer is represented by G4 PAMAM-OH-(Pt^2+^)_*n*_-(Indo)_*m*_, as represented in Scheme  2(c). The indomethacin-dendrimer mixture was vortexed for 30 min. and allowed to gestate for an additional 2-3 days [10]. 

 For the surface deposition of dendrimers, as shown in Scheme  2(d), 1 cm^2^ pieces of gold films were H_2_-flamed [34] and allowed 20 min cooling under clean ambient conditions. Then, a ~75.0 *μ*L drop of G4 PAMAM-OH-(Pt^2+^)_*n*_-(Indo)_*m*_ dendrimer solution was deposited onto the Au(111) surface and allowed to contact for 1.25 min. After washing with water and ethanol the surface was flooded with a 1.0 mM C_8_ solution for 2 min. The surface was then washed again with ethanol and dried under N_2_ before STM and AFM imaging. The formation of C_8_ SAMs confines dendrimers laterally, thus maintaining the structural integrity, and prevents lateral movement during scanning [28]. SAMs also serves as an important internal reference standard for lateral calibration.

### 3.4. Combined AFM and STM Investigations Enable the Size and Geometry of Individual Dendrimers to be Determined

**Scheme 3:**
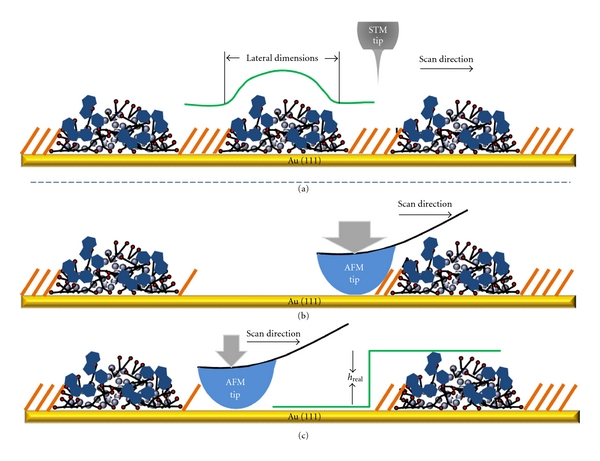
Method of measuring the volume of PAMAM dendrimers using STM and AFM. The *h*_APP_ and lateral dimensions of single dendrimers are obtained through STM topographs (a). The removal of surface adsorbates under high force (b) allows for AFM height measurements during subsequent scans under normal imaging load (c).

 While STM enables high-resolution imaging and accurate determination of the lateral dimension of individual dendrimers [28], AFM allows for the height to be measured precisely [28, 45, 46].  Scheme 3 illustrates this combined approach. In STM imaging, the tip is located at a fraction of a nanometer above the surface (green tracking line). The current between the W-probe and Au surface is the feedback signal and very localized, and as such, the lateral dimension of the features (e.g., dendrimers) underneath are clearly defined from topographic images. The height in the STM topograph is influenced by the local structure as well as local density of states (LDOS). Although the STM height, referred to as apparent height (*h*_APP_), is a sensitive indicator of surface features, the accuracy is difficult to gauge due to the difficulties in determining the LDOS contribution. Therefore, AFM is frequently utilized for the same sample to determine the height of dendrimers [28]. As illustrated in Scheme 3, the true height of the PAMAM dendrimers is measured from the Au substrate to the apex of the dendrimer. For the cleanness of the Au substrate, nanoshaving is exercised to remove adsorbates from the defined area to expose the Au as a reference of the origin [28]. Our previous studies have correlated the *h*_APP_ and true height with this combined approach [28, 29].

### 3.5. Uploading of Indomethacin Results in Increased Integrity and Size of PAMAM Dendrimers

**Figure 2:**
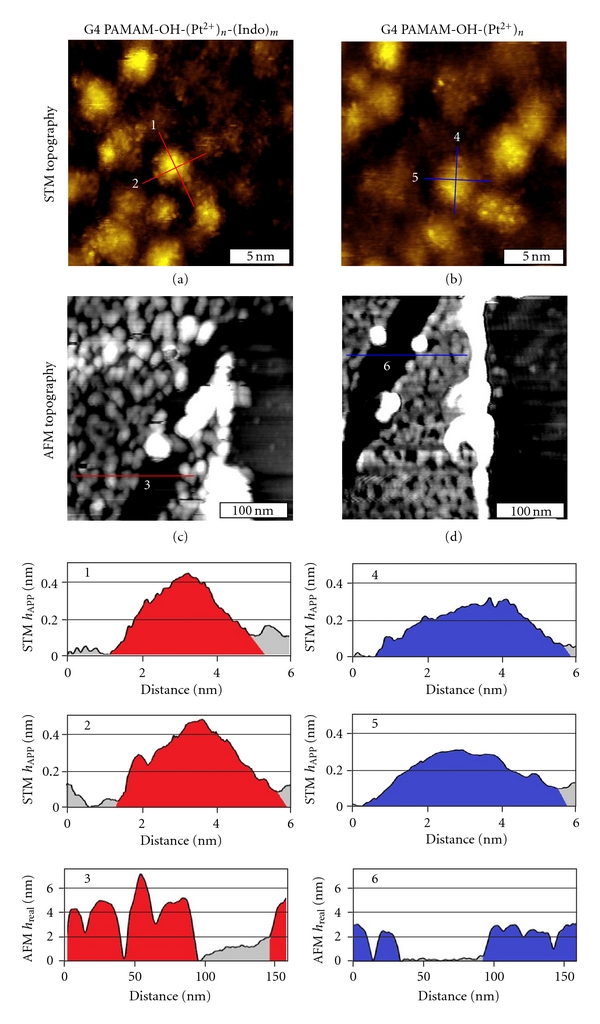
STM and AFM topographic lateral and height measurements determine the volume of single G4 PAMAM-OH-(Pt^2+^)_*n*_-(Indo)_*m*_ and G4 PAMAM-OH-(Pt^2+^)_*n*_ dendrimers. (a) A 20 × 20 nm^2^ STM topograph of G4 PAMAM-OH-(Pt^2+^)_*n*_-(Indo)_*m*_ dendrimers immobilized on Au(111). Cursors 1 and 2 reflect the *h*_APP_ and lateral measurements. (b) A 20 × 20 nm^2^ STM topograph of G4 PAMAM-OH-(Pt^2+^)_*n*_ dendrimers immobilized on Au(111). Cursors 4 and 5 reflect the STM height and lateral measures. STM images (a) and (b) were obtained using circa 0.3 V and 20 pA set points. (c) A 300 × 300 nm^2^ AFM topograph of the same surface as (a). Cursor 3 is a representative of the cursors used to ascertain *h*_REAL_ from the bare Au surface to the dendrimer apex. (d) A 300 × 300 nm^2^ AFM topograph of the same surface as (b). Cursor 6 serves the same purpose for G4 PAMAM-OH-(Pt^2+^)_*n*_ as cursor 3 does for drug-loaded dendrimers.

Figure 2 shows STM images of dendrimers on surfaces. Upon immobilization, dendrimers deform and adopt elliptical cap geometry. Upon uptake of indomethacin, STM imaging reveals that G4 PAMAM-OH-(Pt^2+^)_*n*_-(Indo)_*m*_ dendrimers are taller than the bare G4 dendrimers. Figure  2(a) is a 20 × 20 nm^2^ STM topograph of G4 PAMAM-OH-(Pt^2+^)_*n*_-(Indo)_*m*_ dendrimers immobilized on a Au(111) surface. The bright protrusions correspond to individual G4 PAMAM-OH-(Pt^2+^)_*n*_-(Indo)_*m*_ dendrimer molecules. The STM apparent height, or *h*_APP_, is obtained by measuring the height from the lowest point in the immediate surrounding matrix to the top of the dendrimer. These cursors indicate that dendrimers loaded with indomethacin adopt an elliptical dome shape similar to the base dendrimers reported previously [28, 29, 47, 48]. The *h*_APP_ in cursor profiles 1 and 2 is 0.43 and 0.48 nm, respectively. In contrast, the *h*_APP_ of a typical G4 dendrimer, as shown in Figure  2(a), measures 0.35 nm and 0.33 nm, respectively. The uptake of indomethacin increases the *h*_APP_ by 0.08 nm. Among the 102 dendrimers we compared, drug-loaded G4 dendrimers appear 0.09 ± 0.02 nm taller than the dendrimers themselves. The true height is further investigated using AFM as described in the previous section. The typical real height (*h*_REAL_) for G4 and indo-G4 complexes are 2.5 ± 0.3 nm and 3.4 ± 0.7 nm, respectively. 

 After measuring the lateral and vertical dimensions, the volume of dendrimers can be accurately determined and compared. Assuming an elliptical cap geometry for all dendrimers, the volume of individual molecules may be calculated using *V* = (1/6*πh*_REAL_)(3/4*ab* + *h*_REAL_^2^), where *a* and *b* are the long and short lateral axes, respectively. In a typical case shown in Figure 2, the lateral dimensions are *a* = 5.6 nm, *b* = 4.2 nm for the indomethacin-loaded G4 and the height is 3.4 nm, thus *V* = 52.3 nm^3^. From Figure  2(b), the bare G4 dendrimers measure *a* = 5.7 nm, *b* = 5.2 nm, and *h*_REAL_ = 2.2 nm, which corresponds to a *V* = 31.2 nm^3^. Among the 102 dendrimers compared, drug-loaded dendrimers are 54% more voluminous than base dendrimers. The average lateral dimensions are *a* = 6.8 ± 1.2 nm and *b* = 5.6 ± 0.9 nm for indomethacin-loaded G4 and *a* = 6.2 ± 0.8 nm and *b* = 5.1 ± 0.7 nm for unloaded G4. Since the lateral deformation of both loaded and unloaded G4 dendrimers are similar, the height, and thus volume, increases observed with the addition of indomethacin suggest that the addition of indomethacin to the exterior of dendrimers increases the overall structural integrity upon surface immobilization.

### 3.6. STM Imaging Enables Visualization of Individual Indomethacin Molecules Carried by Dendrimers

**Figure 3:**
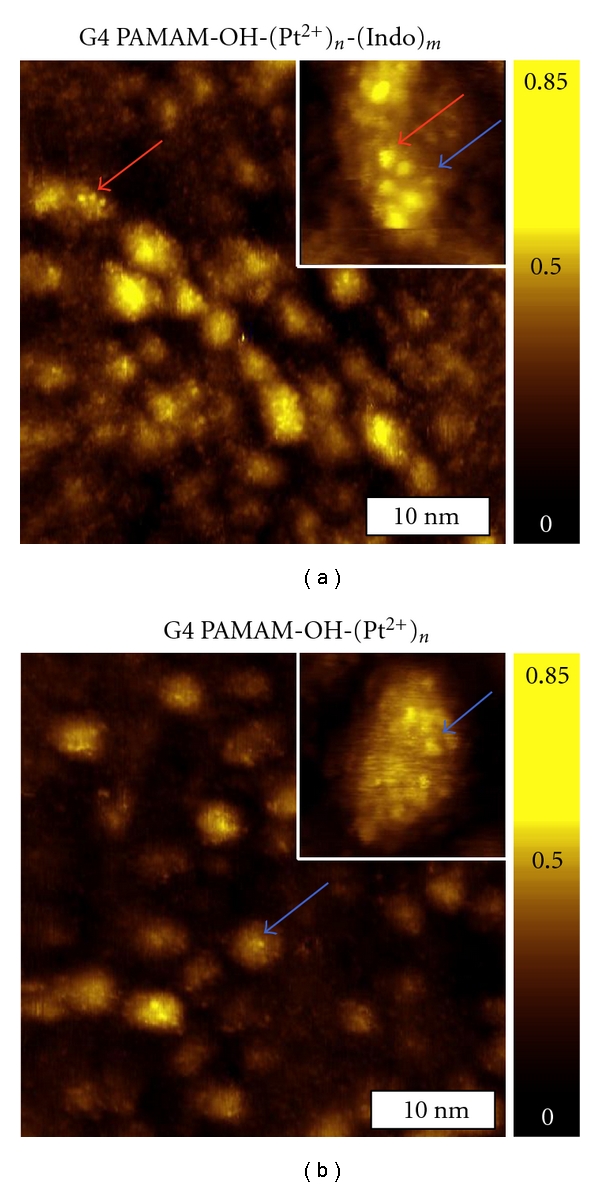
Visualization of indomethacin features from high-resolution STM images. (a) A 40 × 40 nm^2^ STM topographic image of G4 PAMAM-OH-(Pt^2+^)_*n*_-(Indo)_*m*_ dendrimers immobilized on Au(111). The inset in (a) is a 5 × 5 nm^2^ high-resolution image of a single drug-loaded dendrimer from the same surface showing indomethacin features (red arrows) and a dendrimer intramolecular feature (blue arrow). (b) A 40 × 40 nm^2^ STM topographic image of G4 PAMAM-OH-(Pt^2+^)_*n*_ dendrimers immobilized on Au(111). The 6 × 6 nm^2^ inset is a bare dendrimer, where only intramolecular features are visible (blue arrows). All STM images were acquired at 0.3 V and 20 pA. The color scale is normalized to reflect the apparent height range of 0.00–0.85 nm.

 The indomethacin is distinctly recognizable in STM topographs, because they appear taller and usually broader than the −OH termini of dendrimers.  Figure 3 illustrates how to distinguish the two types of features. Since Figures 3(a) and 3(b) display with the same STM apparent height range, the contrast indicates the height and enables a directly comparison. It is clearly seen that the fine features at the surface of G4 PAMAM-OH-(Pt^2+^)_*n*_-(Indo)_*m*_ dendrimers (Figure 3(a)) appear brighter than the unloaded dendrimers which have only −OH at the termini (Figure 3(b)). Figure 3(a) is a STM topographic image of a G4 PAMAM-OH-(Pt^2+^)_*n*_-(Indo)_*m*_ dendrimer surface and the inset is a high-resolution image of a single dendrimer in which the intramolecular features are clearly visible. At first glance, these intramolecular features fall into two types of contrast; that is, one appears brighter than the other. Both previous and present studies of G4-dendrimer reveal the apparent height of −OH termini to be below 0.11 nm [28, 29]. Therefore, we conclude that the bright and tall features identified in Figure 3(a) are new entities, that is, due to attachment by indomethacin. Among 20 G4 PAMAM-OH-(Pt^2+^)_*n*_-(Indo)_*m*_ dendrimers analyzed, indomethacin features have a *h*_APP_ range = 0.12–0.25 nm with an average *h*_APP_ = 0.16 nm. In contrast, intramolecular feature *h*_APP_ measured on indomethacin-loaded and unloaded metal ion-doped G4 PAMAM-OH dendrimers ranged 0.03–0.10 nm, among 40 dendrimers measured previously [28] and in this study. Using the threshold of 0.12 nm, we were able to assign intramolecular and indomethacin features in the STM images, therefore, to count how many indomethacin each dendrimer could carry. Among all 19 intramolecular protrusions visible in Figure 3(a), 13 fall under 0.12 nm (0.03 to 0.11 nm), and 6 are above 0.12 nm (0.13 to 0.17 nm), thus assignment of 13 termini and 6 indomethacin molecules. Figure 3(b) is an STM topographic image of a base dendrimer molecule, G4 PAMAM-OH-(Pt^2+^)_*n*_, where intramolecular features, or −OH termini, are clearly visible [28]. The number of indomethacin molecules carried by G4 PAMAM-OH varies from 2 to 14 among the 20 typical dendrimers analyzed in this investigation. This range is consistent with a previous report where each G4 PAMAM-OH dendrimer molecule could hold 12.5 indomethacin [11]. It is possible that indomethacin may reside in the dendrimer interior void space; therefore, the observed number of indomethacin per dendrimer most likely represents the minimum uptake. Our investigations also reveal that the drug carrying capacity (load) increases with the generation, for example, G3, G4, and G5 PAMAM-OH-(Pt^2+^)_*n*_-(Indo)_*m*_ dendrimers carry 5–7, 2–14, and 2–19 drugs, respectively [29]. The variations in height and geometry of dendrimer-immobilized indomethacin molecules suggest that drugs are nonspecifically bound to the dendrimer termini and exposed amidoamine moieties. 

## 4. Conclusions

This study demonstrates the significance of using STM and AFM in the fundamental studies of new drug-delivery vehicles, telodendrimer micelles and PAMAM dendrimers. The preliminary results indicate that the exquisitely high-resolution images enable insightful and fundamental information be revealed in the context of molecular level location and load of drug molecules, as well as the stability of drug-carrier complex. The number of drug molecules per carrier can be directly extracted in the case of dendrimers and estimated in the case of telodendrimer micelles. Since those studies are at the individual carrier's level, the results can be directly linked to simulations which shall facilitate the prediction and design of new carriers.
